# Bovine tuberculosis slaughter surveillance in the United States 2001–2010: assessment of its traceback investigation function

**DOI:** 10.1186/s12917-014-0182-y

**Published:** 2014-08-15

**Authors:** Heather M Humphrey, Kathleen A Orloski, Francisco J Olea-Popelka

**Affiliations:** 1 Department of Clinical Sciences, College of Veterinary Medicine and Biomedical Science, Colorado State University, Fort Collins 80523, Colorado, USA; 2 Animal and Plant Health Inspection Service (APHIS), United States Department of Agriculture (USDA), Fort Collins 80523, Colorado, USA; 3 Mycobacteria Research Laboratories (MRL), College of Veterinary Medicine and Biomedical Science, Colorado State University, Fort Collins 80523, Colorado, USA

**Keywords:** Bovine tuberculosis, Cattle, Slaughter surveillance, Traceback

## Abstract

**Background:**

The success of tracing cattle to the herd of origin after the detection and confirmation of bovine tuberculosis (TB) lesions in cattle at slaughter is a critical component of the national bovine TB eradication program in the United States (U.S.). The aims of this study were to 1) quantify the number of bovine TB cases identified at slaughter that were successfully traced to their herd of origin in the U.S. during 2001–2010, 2) quantify the number of successful traceback investigations that found additional TB infected animals in the herd of origin or epidemiologically linked herds, and 3) describe the forms of animal identification present on domestic bovine TB cases and their association with traceback success.

**Results:**

We analyzed 2001–2010 data in which 371 granulomatous lesions were confirmed as bovine TB. From these 114 bovine TB cases, 78 adults (i.e. sexually intact bovines greater than two years of age), and 36 fed (i.e. less than or equal to two years of age) were classified as domestic cattle (U.S. originated). Of these adults and fed cases, 83% and 13% were successfully traced, respectively. Of these traceback investigations, 70% of adult cases and 50% of fed cases identified additional bovine TB infected animals in the herd of origin or an epidemiologically linked herd. We found that the presence of various forms of animal identification on domestic bovine TB cases at slaughter may facilitate successful traceback investigations; however, they do not guarantee it.

**Conclusions:**

These results provide valuable information with regard to epidemiological traceback investigations and serve as a baseline to aid U.S. officials when assessing the impact of newly implemented strategies as part of the national bovine TB eradication in the U.S.

## Background

A program to control and eradicate bovine tuberculosis (TB) in cattle was initiated by the United States (U.S.) government in 1917 [1]. Today, the TB Eradication Program is a cooperative effort among the U.S. Department of Agriculture (USDA) Animal and Plant Health Inspection Service (APHIS), Food Safety and Inspection Service (FSIS), State animal health agencies and U.S. livestock producers [2],[3]. In 2009, bovine TB herd prevalence in the U.S. had decreased to an estimated 1 per 100,000 (0.001%) and in cattle had decreased to an estimated 0.1 per 1,000,000 (0.00001%) [4]. Despite the low prevalence and the program’s sustained efforts, the goal of eradication has remained elusive. Bovine TB continues to be detected sporadically in U.S. livestock herds and remains a serious and costly disease of livestock in the U.S. [3],[5]. Between 1998 and 2010, nine States across the country (CO, CA, TX, NE, MN, MI, NM, IN, and NY) detected bovine TB in at least 82 cattle herds and 10 captive cervid herds [6].

During the first half of the twentieth century, the program emphasized a stringent test and slaughter strategy of control, involving systematic and routine farm-to-farm area wide testing of cattle using the tuberculin skin test and the slaughter of all reactors [7]-[9]. The program made considerable progress with this approach and the disease prevalence in cattle herds decreasing from 4.9% in 1918 to 0.3% in 1941 [3]. By 1941, every county in the U.S. had achieved “Modified Accredited” status signifying that the prevalence in the area was below 0.5%^a^[8],[10],[11].

Around 1960, the emphasis of the program shifted from routine area wide tuberculin testing to slaughter surveillance (meat inspection) combined with traceback investigations [1],[12],[13]. The official process for conducting TB slaughter surveillance in regular kill cattle in the U.S. involves the incision and inspection of lymph nodes in the head and chest cavity for granulomas. Other lymph nodes, such as those in the abdomen, may be inspected for other reasons. All head and thoracic granulomas, as well as all other granulomatous lesions regardless of anatomical location, are submitted [14]. It is important to mention that slaughter surveillance (post-mortem examination) has a low sensitivity for detecting all animals with bovine TB lesions [15]-[18]. Thus, once a bovine TB case is found and confirmed, it is crucial for the goals of a national bovine TB control program to identify the source (herd) of that case.

FSIS veterinarians who identify a granulomatous lesion suggestive of bovine TB, submit the lesion, along with VS Form 6–35 “Report of Tuberculosis Lesions or Thoracic Granulomas in Regular Kill Animals” and any available animal identification (ID), to federally approved laboratories for analysis. Currently, histopathology, polymerase chain reaction (PCR) and bacteriological culture are performed on the submitted tissue to confirm bovine TB [14],[15]. If a lesion is found to be histopathologically compatible for mycobacteriosis, State or Federal animal health officials begin a traceback investigation [19]. The most often isolated *Mycobacterium* species from a lesion compatible for mycobacteriosis is *Mycobacterium bovis (M. bovis)*. If additional laboratory results indicate that the pathogen is not *M. bovis*, the investigation stops and does not progress.

A traceback investigation is the process of tracing a bovine TB case from slaughter back to the herd of origin in the U.S. The purpose of a traceback investigation is to find the herd of origin and related herds (animals at risk of having been exposed to *M. bovis*). To find the herd of origin and related herds, all of the available receipts and records detailing the infected animal’s movements from various owners, livestock markets and/or feedlots and states (using the interstate certificates of veterinary inspection (ICVI)) are reviewed. Upon finding the herd of origin, cattle are tested using the caudal fold tuberculin test (CFT) followed by the comparative cervical tuberculin (CCT) test or gamma interferon (GI) assay. Based on the strength and accuracy of the information leading to the herd of origin, CFT responders are slaughtered and necropsied (strong evidence) or administered a secondary test (uncertain evidence) [19]. The secondary test choice is based primarily on logistical considerations of samples reaching the laboratory within 24 hours of collection for the GI assay. When this is not possible, the CCT test is used [19],[20]. The CCT test or the GI assay are used to rule out false positives (series interpretation). Based on USDA TB program requirements, test animals classified as positive are euthanized and tissues tested by histology and culture. In addition, tissues with a histologic diagnosis consistent with mycobacterial infection are tested by PCR [15],[19]. Cattle that have been sold out of a known affected herd, prior to the herd infection being detected are considered exposed [19]. If *M. bovis* is confirmed in a herd, the subsequent investigation may include identification of all epidemiologically linked (contact) herds: adjacent (immediate neighboring) and surrounding herd(s) (in the vicinity), trace-ins to find the source of infection, and trace-outs through registered sales and livestock auction markets to find other exposed animals and herds [3],[20]. These epidemiological investigations evaluate movement of cattle up to five years previous to the time of identification of an affected herd [3]. Upon finding epidemiologically linked herds, cattle are tested using the CFT test followed by the CCT test or the GI assay [20].

Since the emphasis of the bovine TB control program in the U.S. shifted to slaughter surveillance, traceback investigations are the primary method by which the USDA/APHIS identifies herds in the U.S. infected with bovine TB. For example, in 2005, approximately, 95% of the TB infected herds were detected through slaughter traceback and subsequent epidemiological investigations [7]. Thus, the submission of potentially tuberculous lesions for laboratory examination and the success and timeliness of traceback investigations to the herd of origin are crucial for disease control and the success of the program [1].

In several publications concerns have been expressed about the effectiveness of tracing bovine TB cases from slaughter back to the herd of origin in the U.S. [2],[9],[21]-[24]. In 2009, the USDA described eight major challenges for the eradication of bovine TB in U.S. national cattle herd. One of the challenges was the “inability to trace some infected animals identified at slaughter back to a herd” [5]. Kaneene et al. [2] suggested that the “success rate” of tracing bovine TB infected cattle back to a herd of origin was between 50 and 70% of the investigations undertaken. To date, however, a formal study has not been conducted in the U.S. to determine the proportion of traceback investigations that successfully traced a bovine TB case detected during slaughter surveillance back to the U.S. herd of origin. Thus, we conducted this study with the purpose to assess the ability of the current bovine TB slaughter surveillance system to trace confirmed bovine TB cases back to the herd of origin in the U.S. The specific objectives for this study were to 1) quantify the number of investigations that successfully traced bovine TB cases to their herd of origin in the U.S. during 2001–2010, 2) quantify the number of successful traceback investigations that found additional bovine TB infected animals in the U.S., and 3) describe the forms of animal ID present on the domestic bovine TB cases at slaughter and their association with traceback success.

## Methods

### Data sources

Data was obtained from USDA/APHIS/Veterinary Services (VS) and consisted of an electronic spreadsheet containing information on all lesions found at slaughter that were confirmed to be bovine TB. The only three laboratories that received and processed samples were the National Veterinary Services Laboratories (NVSL), Iowa, the Food Safety and Inspection Services laboratory, Georgia, and the California Animal Health and Food Safety Laboratory.

The data set analyzed contained laboratory test results (histopathology, PCR and culture), age and sex of the animal if known, forms of animal ID if present, country or state of origin if known, brief descriptive comments regarding the investigation status, and whether the investigation was completed or ongoing. In addition, supporting documents, i.e., case closing reports, miscellaneous case notes and emails, tuberculin test reports, NVSL laboratory reports, and an affected herd spreadsheet, were available for each bovine TB case either in paper or electronic format.

### Inclusion criteria

Only bovines confirmed to have bovine TB were included in this study. Confirmation of bovine TB was achieved when histology was compatible for mycobacteriosis and PCR was positive for *Mycobacterium tuberculosis* complex or *M. bovis* was isolated during culture of submitted tissue samples. Laboratory results not meeting these criteria resulted in the case being excluded. For the purposes of determining traceback success, cases in cattle imported into the U.S. were excluded.

### Outcome definition

The first outcome measured was success (or failure) of tracing bovine TB cases from slaughter back to the herd of origin in the U.S. It is worth noting that for the purposes of this study a successful traceback investigation was classified when the most recent herd of origin in which the animal with a confirmed bovine TB lesion resided was found. A herd of origin was defined as a group of breeding livestock in the U.S. Feedlots, dealers, and calf- or heifer-raising facilities were excluded. For example, if a culled adult cow was placed in a feedlot for several weeks subsequent to leaving its most recent breeding herd of residence and prior to being slaughtered, tracing to the feedlot only was not classified as a successful trace.

The second outcome measured in this study was determining if bovine TB infection was confirmed in the herd of origin after it was found or in an epidemiologically linked herd(s) (identified through secondary trace-in or trace-out investigations) in the U.S. When bovine TB is detected through slaughter surveillance, the USDA requires confirmation of bovine TB in the herd from which the slaughter case originated to consider the herd as infected. Confirmation is achieved by detecting at least one additional bovine TB infected animal in the herd, as a result of testing the herd with antemortem tests [19]. Additional affected herds may be identified through further epidemiological investigations using previously described testing protocols [2],[14],[19].

#### Country of origin of bovine TB cases

Federal and State animal health officials use information from a variety of sources to determine the country of origin for each bovine TB case identified at slaughter. All available information at the time of slaughter (slaughter plant kill sheets, contact information for consignors for the slaughter lot where the animal resided, completed VS Form 6–35, any animal ID) and during the traceback investigation (e.g., bill of sale receipts, records and documentation from producers, dealers/brokers, livestock markets, and slaughter plants, interstate certificates of veterinary inspection (ICVI), importation documents and related documents) were used to determine the country of origin of bovine TB cases.

Bovine TB cases found at slaughter could have had management ID (farm specific ID; not country or state specific), a U.S. form of animal ID (brucellosis vaccination tag, USDA backtag, and/or National Uniform Eartagging System tag (also known as a NUES or brite tag)), Mexican ID or Canadian ID. None of the bovine TB cases had Animal Identification Number (AIN) “840” tags. Some cases had no ID. Bovine TB cases were considered U.S. by animal health officials if they had U.S. ID such as a brucellosis vaccination tag, USDA backtag, and/or NUES (brite) tag or the traceback investigation determined they were U.S. Cattle were determined to be of Mexican origin if they had an official Mexican ID or the traceback investigation determined they were Mexican. Cattle were identified as Canadian origin if they had official Canadian ID or the traceback investigation determined they were Canadian. Cattle that lacked U.S. ID or official Mexican or Canadian ID were pursued with diligence by animal health officials during the traceback investigation. Animal health officials were often able to determine whether an animal was “most likely” imported and these cases were classified as such. For example, a bovine TB case without animal ID was determined to be of Mexican origin when the consignor of the cattle affirmed that only Mexican cattle were present in the lot of cattle that were slaughtered. For the purposes of this analysis, bovine TB cases that had official Mexican or Canadian ID and any bovine TB case that was determined by Federal and State animal health officials to be “most likely” Mexican or Canadian were classified as imported and thus, considered as not having a herd of origin in the U.S. The remaining cases were classified as domestic, potentially having a herd of origin in the U.S. One hundred and fifty seven bovine TB cases had official Mexican ID, two bovine TB cases had official Canadian ID, 97 bovine TB cases were determined to be “most likely” of Mexican origin and one bovine TB case was determined to be “most likely” of Canadian origin. These 257 bovine TB imported cases were excluded from the analysis conducted to assess the ability of the current bovine TB slaughter surveillance system to trace bovine TB cases back to the herd of origin in the U.S. Factors related to these imported bovine TB cases impacting the epidemiology of the disease in the U.S. are briefly discussed. The remaining 114 bovine TB cases were classified as domestic for our analysis, thus considered having a herd of origin in the U.S.

### Analysis

**Figure 1:**
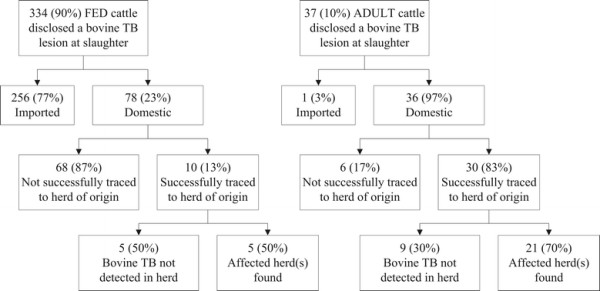
**Distribution by age of successful traceback investigations to a herd of origin in the U.S. and number that yielded at least one affected herd, 2001–2010.** Of the 334 fed bovine TB cases identified at slaughter between 2001–2010, the majority (256, 77%) were classified as imported animals (254 from Mexico and 2 from Canada). Traceback to a herd of origin for these animals was beyond the scope of this study. Seventy-eight (23%) were determined to be domestic, potentially having a herd of origin in the U.S. From these domestic fed bovine TB cases, 10 (13%) were successfully traced to a herd of origin in the U.S. and 68 (87%) cases were not. As part of the traceback investigations on the 10 domestic fed bovine TB cases identified at slaughter that were successfully traced to a herd of origin in the U.S., in 5 of them additional infected animals (affected herds) were identified when either the herd of origin or an epidemiologically linked herd (identified through secondary trace-in or trace-out investigations) were tested using the official bovine TB program tests. Of the 37 adult bovine TB cases identified at slaughter between 2001–2010, 1 was determined to be imported (from Canada) and 36 (97%) domestic. From these domestic adult bovine TB cases, 30 (83%) were successfully traced to a herd of origin in the U.S. and 6 (17%) were not. As part of the traceback investigations on the 30 domestic adult bovine TB cases identified at slaughter that were successfully traced to a herd of origin in the U.S., in 21 (70%) of these additional infected animals (affected herds) were identified when either the herd of origin or an epidemiologically linked herd (identified through secondary trace-in or trace-out investigations) were tested using the official bovine TB program tests.

Descriptive statistics were performed in order to quantify the number of domestic bovine TB cases that had a successful traceback investigation to the herd of origin in the U.S. (objective 1) and the number of successful investigations that led to finding additional bovine TB infected animals (“affected herds”) in the U.S. (objective 2). The findings were summarized as proportions (Figure 1). Information from each confirmed bovine TB case were obtained by review of the case closing reports, miscellaneous case notes, the tuberculin test record reports, NVSL laboratory reports, the affected herd spreadsheet and evaluation of each epidemiological investigation. Results are tabulated based on two age categories of the cattle with bovine TB lesions: fed and adult. Fed animals were bovines less than or equal to two years of age, while adult animals were sexually intact bovines greater than two years of age. Fed and adult bovine TB cases are deemed, by Federal and State animal health officials, to pose different epidemiological risks to the national herd. Different forms of animal ID present among domestic bovine TB cases are summarized and presented in Table 1 which includes the success of the traceback investigation. Excel^b^ was used for the descriptive analyses.

**Table 1:** Presence of animal ID and traceback success for domestic cattle disclosing lesions at slaughter, 2001–2010

**Presence or absence of animal identification (ID) at slaughter**	**Successfully traced**	**Not successfully traced**	**Total number of cases**
Domestic fed bovine TB cases			
A U.S. form of ID and management ID	1	0	1
A U.S. form of ID	0	2	2
Management ID	6 (11%)	47 (89%)	53
No ID	3 (15%)	19 (86%)	22
Total	10 (13%)	68 (87%)	78
Domestic adult bovine TB cases			
A U.S. form of ID and management ID	5 (83%)	1 (17%)	6
A U.S. form of ID	16 (84%)	3 (16%)	19
Management ID	4	0	4
No ID	5 (71%)	2 (29%)	7
Total	30 (83%)	6 (17%)	36
Overall domestic (fed & adult) bovine TB cases			
A U.S. form of ID and management ID	6 (86%)	1 (14%)	7
A U.S. form of ID	16 (76%)	5 (24%)	21
Management ID	10 (18%)	47 (82%)	57
No ID	8 (30%)	21 (72%)	29
Grand Total	40 (35%)	74 (65%)	114

The findings of the forms of animal ID present at slaughter among the 114 domestic bovine TB cases and the outcome of the investigation with regard to tracing back to the herd of origin in the U.S. Domestic bovine TB cases found at slaughter had no animal ID or one or more forms of the following ID: management ID (farm specific ID; not country or state specific) and/or a U.S. form of ID (brucellosis vaccination tag, USDA backtag and/or NUES tag).

## Results

During 2001–2010, a total of 374 animals were confirmed as infected with bovine TB after disclosing a lesion at slaughter. Of these, 371 were bovines and three were cervids. The majority (n = 334, 90%) occurred in fed cattle, and 37 cases (10%) occurred in culled adult cattle.

### Distribution by age of successful traceback investigations to a herd of origin in the U.S. and number that yielded at least one affected herd, 2001–2010

Of the 334 bovine TB cases in fed cattle, 256 (77%) occurred in imported animals, including 254 from Mexico and 2 from Canada (Figure 1). The remaining 78 cases (23%) were classified as domestic cattle. Of these domestic fed cases, only 10 (13%) were successfully traced to a herd of origin in the U.S., and for five of these herds, additional infected animals were identified by antemortem TB testing in the herd of origin or epidemiologically linked herd (Figure 1).

Of the 37 adult bovine TB cases identified at slaughter, 36 cases were domestic (Figure 1). One case occurred in an animal imported from Canada. Of these 36 domestic cases, 30 (83%) were successfully traced to a herd of origin in the U.S. In 21 herds (70%), additional infected animals (affected herds) were identified in either the herd of origin or an epidemiologically linked herd (identified through secondary trace-in or trace-out investigations) using official bovine TB program tests (Figure 1).

### Summary of animal ID forms present at time of slaughter and traceback success for domestic bovine TB cases

Table 1 summarizes the forms of animal ID present at slaughter among the 114 domestic bovine TB cases and the outcome of the traceback investigation with regard to successfully tracing to the herd of origin in the U.S. Forms of animal ID found among the domestic bovine TB cases was variable and consisted of a U.S. form of ID (brucellosis vaccination tag, USDA back tag, and/or NUES (brite) tag) and management ID. The majority (53%, 19/36) of domestic adult bovine TB cases had a U.S. form of ID, while the majority (68%, 53/78) of domestic fed bovine TB cases had management ID.

The results by age show that of the 78 domestic fed bovine TB cases, 10 cattle were successfully traced to the herd of origin. Only one domestic fed bovine TB case had a U.S. form of ID and management ID and was successfully traced. The two domestic fed bovine TB cases that had a U.S. form of ID were not successfully traced. The majority (89%, 47/53) of domestic fed bovine TB cases with management ID were not successfully traced; only six (11%) domestic fed bovine TB cases with management ID were successfully traced. The majority (86%, 19/22) of domestic fed bovine TB cases without animal ID were not successfully traced. Three of the 22 (15%) domestic fed bovine TB cases without animal ID were successfully traced.

Of the 36 domestic adult bovine TB cases, 30 cattle were successfully traced. From the six domestic adult bovine TB cases that had a U.S. form of ID and management ID, five were successfully traced and one was not successfully traced. Also, the majority (84%, 16/19) of domestic adult bovine TB cases that had a U.S. form of ID were successfully traced; three (16%) domestic adult bovine TB cases with a U.S. form of ID were not successfully traced. All of the four domestic adult bovine TB cases that had management ID were successfully traced. The majority (71%, 5/7) of domestic adult bovine TB cases without animal ID were successfully traced, indicating that traceback investigations were conducted successfully with no animal ID. Two (29%) domestic adult bovine TB cases without animal ID were not successfully traced.

## Discussion

Slaughter surveillance and its associated traceback investigations play a crucial role in the U.S. bovine TB eradication program because it is the primary tool for identifying bovine TB cases and infected herds [1],[25]. The results of our study confirm the concerns previously expressed by other authors [2],[9],[21]-[24] with regard to the ability to trace confirmed bovine TB cases from slaughter to their herd of origin in the U.S. The overall proportion of bovine TB cases successfully traced back to a herd of origin (35%) found in our study (83% and 13% for adult and fed cases respectively) is lower than the 50-70% success “rate” cited by Kaneene et al. [2] and is lower than other countries. For example, Mexico reported 80.5%, 89.55%, and 90.29% success in tracing bovine TB cases from slaughter back to the herd of origin for 2009, 2010, and 2011, respectively (Reyes, J.A.G. 2011. Unpublished observations. Mexico National Tuberculosis Report. SAGARPA/SENASICA. 115th Annual Meeting of the United States Animal Health Association. Oct 4. Buffalo, NY). In the Republic of Ireland during the year 2003, all the bovine TB cases identified at slaughter were successfully traced to the herd of origin [26]. This level of success can be achieved in the Republic of Ireland due to a fully implemented animal identification and management system [26].

The majority of bovine TB cases (334, 90%) identified between 2001–2010 were fed cattle. Seventy-eight of these fed bovine TB cases were considered to be domestic and 13% were successfully traced to the herd of origin. It is important to note that fed cattle, with their short lifespan of 24 months or less, are perceived to present minimal risk for spreading infection to other animals, particularly to domestic breeding cattle. However, the detailed review of case investigations of bovine TB infected cattle performed during this study revealed multiple opportunities for exposure to breeding cattle, from feedlots to pasture situations, as did an assessment performed in 2011 by USDA/APHIS/VS. For example, beef herds have been infected by purchased additions, i.e., young male dairy calves that were grafted onto beef cows, and replacement beef and dairy heifers have been exposed to high-risk feeder cattle in feedlots [3]. Based on animal management practices in the U.S., there is a possibility these fed bovine TB cases were exposed to *M. bovis* earlier in their life at a cow-calf operation, stocker or backgrounding operation where animals not destined for slaughter may be present. An additional concern with not tracing back to cow-calf operations is the fact that the grazing lands (pasture, range land, Federal land) that domestic cows and growing calves are reared on may be adjacent and not separated by fencing, resulting in animals belonging to different owners being comingled [27]. We think that despite the relatively lower risk posed by domestic fed cattle with confirmed bovine TB lesions at slaughter (compared to domestic adult cattle) it is extremely important to maximize efforts during a traceback investigation to successfully identify all of the infected animal’s herds and locations prior to slaughter, in particular back to the cow-calf operation where breeding cattle reside.

Our findings indicate that the percentage of successful traceback was higher for domestic culled adult bovine TB cases (83%) compared to domestic fed bovine TB cases (13%). Compared to domestic fed bovine TB cases, this higher proportion of successful traceback investigations is consistent with Federal and State animal health officials and industry management practices that prioritize tracing domestic adult bovine TB cases because they pose the most risk of disease transmission to other cattle. These cattle have a longer lifespan than fed cattle due to their role in being part of a breeding herd and a higher probability of contact (direct or indirect) with other animals throughout their lifespan. The proportion of successful traceback investigations for domestic adult bovine TB cases (83%; 30/36) is commendable; however, the lack of success in identifying the herd of origin for 6 domestic adult bovine TB cases (17%) hinders U.S. bovine TB eradication efforts.

It is important to note that the majority (256/334; 77%) of fed bovine TB cases identified during 2001–2010 as part of slaughter surveillance in the U.S. were imported cattle (254 from Mexico and 2 from Canada). Bovine TB cases identified among imported cattle were excluded from our analysis. However, while evaluating the 2001–2010 traceback epidemiological investigations, in some cases there were indications that domestic and imported infected cattle had the opportunity for contact (direct or indirect). To mitigate this risk, antemortem TB testing is performed when an investigation determines that cattle have been exposed outside of the feedlot. Thus, we recommend that Federal and State animal health officials maintain rigorous further investigation standards regarding animal management and movements of imported live cattle in order to assess the risk of infection that these imported cattle pose to domestic animals while they reside in the U.S.

The results of our second objective bring to fruition the negative impact of not finding the herd of origin for six adults and 68 fed domestic bovine TB cases found at slaughter. Once the herd of origin of a bovine TB case was identified, overall 65% (26/40) of the traceback investigations found additional bovine TB cases in the herd of origin or epidemiologically linked herd. This finding indicates that failure in finding the herd of origin for bovine TB cases could be a significant constraint in controlling bovine TB in the U.S. since it could represent a missed opportunity to identify additional infected animals and implement control measures. This has a negative effect on the amount of time (months or years) before infected herds may be discovered through slaughter surveillance and delays the eradication of bovine TB from the U.S. This scenario creates additional financial loss to livestock owners whose herds may become infected and necessitates tax dollars be added to the program as a result of the spread of infection.

During our study period, a relatively high within herd prevalence has been found in some investigations that successfully traced back the bovine TB case found at slaughter to the herd of origin [28],[29]. A high within herd prevalence strongly suggests that the disease was present within the herd for a substantial length of time before being identified. Similar scenarios with high within herd prevalence (up to 80% and 70% of the animals tested positive at time of testing in the 1990’s and 1999, respectively) have been reported in the Netherlands, a country considered to be free of bovine TB that also relies on slaughter surveillance as the primary method of detecting disease, complemented with traceback investigations [30],[31]. It was estimated that after introduction of the infection into a herd, the median time until a detection of a bovine TB lesion via visual inspection of carcasses at the slaughterhouse was 302 weeks (approximately 5 years) [31]. The scenarios with high within herd prevalence show the importance of detecting bovine TB as early as possible and the potential implications for a particular herd (and other herds) when surveillance efforts fail to identify infection when it is present. The high proportion of traceback investigations identifying affected herds, in the U.S. after a bovine TB case was identified during slaughter surveillance, is likely the result of the combination of the chronic nature of bovine TB and a time component allowing an effective spread of *M. bovis* both within and between herds. Therefore, when a bovine TB lesion is detected at slaughter in the U.S., it is in the best interest of the country to maximize the ability to find the herd of origin as a means to identify additional infected animals and herds. Failure to identify the herd of origin for all cattle disclosing bovine TB lesions at slaughter will increase the likelihood of infection to remain undetected for years, thus increasing the possibility of spread within and between herds and posing a significant constraint to the eradication of bovine TB from the U.S. In addition, it is important to note that currently, unpasteurized (raw) milk sales are legal in 26 States [32], thus, the presence of undetected infected cows is concerning because *M. bovis* remains as a zoonotic agent posing a public health risk via the consumption of unpasteurized milk or dairy products [33].

While conducting the analysis described in this study, there were challenges determining the country of origin for bovine TB cases due to the nature of the current system for identifying cattle (animal identification forms). The difficulty with using U.S. forms of ID as means of identifying animals of U.S. origin is that these forms of ID, while indicative of nationality of the cattle, are not absolute proof of U.S. origin [34]. For example, a USDA backtag does not necessarily reflect an animal’s country of origin, as these temporary tags are applied at concentration points, such as livestock markets and slaughter establishments, and generally without knowledge of the animal’s birth origin. Other authors [2] have alluded to these types of challenges when conducting this type of analysis. The criteria used in our analysis allowed any bovine TB case with an indication of being imported to be classified as such. When the traceback investigations conducted on these animals did not find any evidence to conclude animals were imported or “most likely imported”, these animals were classified in our analysis as domestic cattle. Our approach was a conservative measure taken to minimize misclassification and ensure imported animals were not misclassified as domestic cattle.

With regard to the forms of animal ID present on domestic bovine TB lesioned cattle identified at slaughter, the overall results (fed and adult combined) indicate that the presence of a U.S. form of ID and management ID (or both) facilitate successful traceback investigations; however, they do not ensure traceback success (Table 1). The majority (6/7) of domestic bovine TB cases that had a U.S. form of ID and management ID were successfully traced (1 bovine TB case with a U.S. form of ID and management ID was not successfully traced). Also, the majority (76%) of domestic bovine TB cases that had a U.S. form of ID were successfully traced (5 domestic bovine TB cases with a U.S. form of ID were not successfully traced). Some (18%) domestic bovine TB cases with management ID were successfully traced but the majority (overall 82%) were not. It is worth noticing that all 4 domestic adult bovine TB cases with only a farm specific management ID were successfully traced back. It is commendable that Federal and State animal health officials are able to successfully trace these high risk animals and shows the dedication and diligence applied by the officials throughout the investigations. Regarding the domestic fed bovine TB cases, the majority (89%, 47/53) of domestic fed bovine TB cases that had management ID were not successfully traced. In addition, two domestic fed bovine TB cases that had a U.S. form of ID also were not successfully traced. This shows that, specifically in domestic fed bovine TB cases, the presence of animal ID (either a U.S. form of ID or management ID) at slaughter, particularly management ID by itself, does not ensure a successful traceback from slaughter to the herd of origin in the U.S.

The majority (72%, 21/29) of domestic bovine TB cases without animal ID present at slaughter were not successfully traced, indicating the absence of animal ID may hinder the success of traceback investigations. Contrary to what might be expected, a few domestic bovine TB cases (5 adult and 3 fed) were conducted successfully without any animal ID. Review of the traceback investigation case files for these cases indicated success was the result of various factors and scenarios. These factors included complete and accurate individual animal receipts and records, using animal characteristics (e.g., live weight, gender, breed and color), relatively few ownership changes from the herd of origin to slaughter, availability of genotyping results (e.g., the strain of *M. bovis* in the slaughtered animal was previously identified in an infected herd), perseverance of personnel conducting the investigation, and producer cooperation for herd testing. Given the challenges, it is remarkable that Federal and State animal health officials are able to successfully trace some cattle (adult and fed) without any animal ID.

Upon review of the case files, we found that the investigation process as it exists today, particularly when cattle lack animal ID, is undeniably labour and time intensive. In 28 cases with complete data on time spent to complete the epidemiological investigation, we found that the average time spent to conduct a traceback investigation was 61.4 days (SD = 72.3 days), median = 39.5 days, with a range from 7 to 335 days.

We also found in our study that the reasons for fed and adult bovine TB cases not being successfully traced back to a herd of origin included: 1) irreconcilable, incomplete, and/or illegible industry (producer, dealer/broker, market, feedlot, slaughter plant) receipts, records and documentation, and/or 2) absent, insufficient or incorrectly correlated animal ID. Each investigation required animal health officials to analyze receipts and records, if available, from multiple premises. In scenarios where the bovine TB lesioned animal’s owner could not be determined, multiple producers were tested with the CFT test at the government’s expense. Having to test multiple herds is inefficient and costly to both the affected producers and government entities. With better capabilities for tracing animals in the U.S., the need to test multiple herds could be reduced. For some cases, U.S. forms of IDs were issued twice or a new ID applied after a change in ownership or when an ID was lost, without maintaining records that allow continuity across ID and owners. These examples illustrate the complexities of record keeping and animal ID and the challenges faced by animal health officials to successfully trace cattle that are born and raised in the U.S. to their herd of origin. Revising the animal identification system in the U.S. to become more uniform, consistent and comprehensive (i.e., applying an official, national form of U.S. ID to cattle at birth of all genders (male, female) and of all types (dairy, beef and rodeo)) would simultaneously facilitate and expedite the identification of domestic cattle and the tracing of infected cattle to their herd of origin and all premises within and across State lines from birth to slaughter, thus allowing the testing of high risk animals and implementation of disease control measures. In the U.S., recent regulatory changes that took effect in March 2013 are increasing the requirements for U.S. ID for cattle and bison moving interstate [35]. Implementation of these new requirements should address some of the challenges found in this study for the period 2001–2010. We recommended further studies to assess and quantify the impact of the new regulatory changes implemented in March 2013 on the ability to successfully trace back bovine TB cases to the herd of origin.

## Conclusions

This study shows that for the period 2001–2010 Federal and State animal health officials were able to trace some domestic bovine TB cases identified at slaughter back to their herd of origin; however, sufficient gaps exist in the current bovine TB surveillance that impair the ability for officials to trace all domestic bovine TB cases. The proportion of successful traceback investigations found in this study is an impediment to the goal of eradication. In order for slaughter surveillance to be an effective tool to eradicate bovine TB, it is crucial that all of the bovine TB cases detected at slaughter are successfully traced to their herd of origin and to all other exposed herds in order to maximize the detection of TB infected herds, thus preventing sources of infection from remaining undetected in the national herd. It would be advantageous to the goals of the national bovine TB eradication program to be able to achieve a higher level of success in tracing confirmed bovine TB cases from slaughter to the herd of origin in the U.S.

## Endnotes

^a^In 1941, bovine TB prevalence of less than 0.5% in an area was called Modified Accredited; however, as of January 1, 2005 the term Accreditation Preparatory is used for this level of bovine TB prevalence in a State/zone [19].

^b^ Microsoft: 2007, Redmond, Washington.

## Competing interests

The authors declare that they have no competing interests.

## Authors’ contributions

Study design/planning: FJOP, KAO; analysis: HMH; interpretation of results: HMH, FJOP, KAO; drafting and critical revision of the manuscript: HMH, FJOP, KAO. All authors read and approved the final manuscript.

## References

[B1] Frye GH Thoen CO, Steele JH Bovine Tuberculosis Eradication: The Program in the United States Mycobacterium bovis Infection in Animals and Humans 1995 1 Iowa State University Press, Ames 119 181

[B2] Kaneene JB Miller R Meyer RB Abattoir surveillance: the U.S. Experience Vet Microbiol 2006 112 273 282 10.1016/j.vetmic.2005.11.018 16326037

[B3] http://www.aphis.usda.gov/animal_health/emergingissues/downloads/bovine_tb_pathways_2009030711.pdf United States Department of Agriculture/Animal and Plant Health Inspection Service/Veterinary Services: **Assessment of pathways for the introduction and spread of**** *mycobacterium bovis* ****in the United States, 2009.** 2011, .

[B4] http://www.aphis.usda.gov/animal_health/animal_diseases/tuberculosis/downloads/tb_erad.pdf United States Department of Agriculture/Animal and Plant Health Inspection Service/Veterinary Services: **Bovine tuberculosis, infected cattle detected at slaughter and number of affected cattle herds, United States, 2003–2009.** *ᅟ* 2009, .

[B5] Proceedings of the One Hundred and Thirteenth Annual Meeting of the United States Animal Health Association:11–14 Oct 2009 2010 Richardson Printing, San Diego

[B6] http://www.agweb.com/article/Bovine_TB_Colorado_Joins_Eight_Other_States_Whove_Found_the_Disease_291557/Merlo C: **Bovine TB: Colorado joins eight other states who’ve found the disease.** *Ag Web Powered Farm J* 2010, .

[B7] http://www.aphis.usda.gov/animal_health/animal_diseases/tuberculosis/downloads/tb_guidebook.pdf United States Department of Agriculture/Animal and Plant Health Inspection Service/Veterinary Services: *Tuberculosis Sample Submission Manual for Meat Inspection Personnel.* Riverdale: 2005. .

[B8] Palmer MV Waters WR Bovine tuberculosis and the establishment of an eradication program in the United States: role of veterinarians Vet Med Int 2011 2011 1 12 http://www.ncbi.nlm.nih.gov/pmc/articles/PMC3103864/http://www.ncbi.nlm.nih.gov/pmc/articles/PMC3103864/10.4061/2011/816345 PMC3103864 21647341

[B9] http://www.regulations.gov/#!documentDetail;D=APHIS-2009-0073-0002 United States Department of Agriculture/Animal and Plant Health Inspection Service/Veterinary Services: **A New approach for managing bovine tuberculosis: veterinary services’ proposed action plan.** 2009, .

[B10] Roswurm JD Ranney AF Sharpening the attack on bovine tuberculosis Am J Public Health 1973 63 10 884 886 10.2105/AJPH.63.10.884 4742399 PMC1775269

[B11] Olmstead AL Rhode PW An impossible undertaking: the eradication of bovine tuberculosis in the United States J Econ Hist 2004 64 3 734 772

[B12] Essey MA Koller MA Status of bovine tuberculosis in North America Vet Microbiol 1994 40 15 22 10.1016/0378-1135(94)90043-4 8073622

[B13] Gilsdorf MJ Ebel ED Disney TW Thoen CO, Steele JH, Gilsdorf MJ Benefit and Cost Assessment of the U.S. Bovine Tuberculosis Eradication Program Mycobacterium bovis Infection in Animals and Humans 2006 2 Blackwell Publishing, Ames 89 99 10.1002/9780470344538.ch11

[B14] http://www.fsis.usda.gov/OPPDE/rdad/FSISDirectives/6240.1Rev1.pdf United States Department of Agriculture/Food Safety and Inspection Service: **Directive 6240.1 (Rev1).** 2009, .

[B15] http://www.aphis.usda.gov/vs/nahss/cattle/tb_2009_evaluation_of_tb_in_accredited_free_states_jan_09.pdf United States Department of Agriculture/Animal and Plant Health Inspection Service/Veterinary Services: **Analysis of bovine tuberculosis surveillance in accredited free states.** 2009, .

[B16] Corner LA Post mortem diagnosis of *Mycobacterium bovis* infection in cattle Vet Microbiol 1994 40 53 63 10.1016/0378-1135(94)90046-9 8073629

[B17] Frankena K White PW O’Keefe J Costello E Martin SW van Grevenhof I More SJ Quantification of the relative efficiency of factory surveillance in the disclosure of tuberculosis lesions in attested Irish cattle Vet Rec 2007 161 679 684 10.1136/vr.161.20.679 18024922

[B18] Olea-Popelka F Freeman Z White P Costello E O’Keefe J Frankena K Martin W More S Relative effectiveness of Irish factories in the surveillance of slaughtered cattle for visible lesions of tuberculosis, 2005–2007 Irish Vet J 2012 65 2 2 5 http://www.biomedcentral.com/content/pdf/2046-0481-65-2.pdf http://www.biomedcentral.com/content/pdf/2046-0481-65-2.pdf 10.1186/2046-0481-65-2 PMC3311595 22289139

[B19] http://www.aphis.usda.gov/animal_health/animal_diseases/tuberculosis/downloads/tb-umr.pdf United States Department of Agriculture/Animal and Plant Health Inspection Service: **Bovine tuberculosis eradication uniform methods and rules, effective January 1, 2005.** 2004, .

[B20] http://www.michigan.gov/documents/emergingdiseases/552_15_tb_testing_cattle_176119_7.pdf United States Department of Agriculture/Animal and Plant Health Inspection Service: **Veterinary services memorandum No. 552.15. Instructions and recommended procedures for conducting tuberculosis tests in cattle and bison, august 2.** *ᅟ* 2006, 1–22. .

[B21] Anderson RJ Is Tuberculosis Gaining on Us? Hoard’s Dairyman 1959 W. D. Hoard & Sons Company, Fort Atkinson, Wisconsin

[B22] Ranney AF National eradication campaign achievements and problems in the U.S.A Proceedings of the 1st International Seminar on Bovine Tuberculosis for the Americas 1970 Animal Health Division, Agricultural Research Service, United States Department of Agriculture, U.S.A 202 214

[B23] http://www.aphis.usda.gov/traceability/downloads/TraceabilityBusinessPlanSept2008.pdf United States Department of Agriculture/Animal and Plant Health Inspection Service: *A Business Plan to Advance Animal Disease Traceability Through the Harmonization of State, Federal, and Industry Programs and Convergence with the National Animal Identification System*, Version 1.0. USDA publication; 2008. .

[B24] Traceability for livestock moving interstate Fed Regist 2011 76 155 50082 50110 http://www.regulations.gov/#!documentDetail;D=APHIS-2009-0091-0001 http://www.regulations.gov/#!documentDetail;D=APHIS-2009-0091-0001

[B25] Gilsdorf MJ Judge L Ebel ED Thoen CO, Steele JH, Gilsdorf MJ Current Challenges to and Impacts on the U.S. National Bovine Tuberculosis Eradication Program: *Mycobacterium bovis* Outbreaks in Alternative Species and Surveillance Performance Mycobacterium bovis Infection in Animals and Humans 2006 2 Blackwell Publishing, Ames 211 225 10.1002/9780470344538.ch20

[B26] Olea-Popelka FJ Costello E White P McGrath G Collins JD O’Keeffe J Kelton DF Berke O More S Martin SW Risk factors for disclosure of additional tuberculous cattle in attested-clear herds that had one animal with a confirmed lesion of tuberculosis at slaughter during 2003 in Ireland Prev Vet Med 2008 85 81 91 10.1016/j.prevetmed.2008.01.003 18314209

[B27] http://www.ers.usda.gov/publications/aer-agricultural-economic-report/aer830.aspx Golan E, Krissoff B, Kuchler F, Calvin L, Nelson K, Price G: **Traceability in the U.S. Food Supply: Economic Theory and Industry Studies.** In Edited by Economic Research Service, U.S. Department of Agriculture, Agricultural Economic Report No. 830; 2004. .

[B28] http://www.usaha.org/Portals/6/Proceedings/USAHAProceedings-2010-114th.pdf United States Animal Health Association: **Report of the Committee on Tuberculosis.** In *Proceedings of One Hundred and Fourteenth Annual Meeting of the U.S. Animal Health Association: 11–17 Nov 2010; Minneapolis.* Edited by Richardson Printing. 2011:545–556. .

[B29] Francisco TI, Orloski KA, Roberts NJ: **Investigation of a**** *mycobacterium bovis* ****outbreak in cattle at a Colorado dairy in 2010.** *J Am Vet Med Assoc* 2014, in press. 10.2460/javma.244.7.805 24649991

[B30] van Asseldonk MAPM van Roermund HJW Fischer EAJ de Jong MCM Huirne RBM Stochastic efficiency analysis of bovine tuberculosis-surveillance programs in the Netherlands Prev Vet Med 2005 69 39 52 10.1016/j.prevetmed.2005.01.012 15899295

[B31] Fischer EAJ van Roermund HJW Hemerik L van Asseldonk MAPM de Jong MCM Evaluation of surveillance strategies for bovine tuberculosis (*Mycobacterium bovis*) using an individual based epidemiological model Prev Vet Med 2005 67 283 301 10.1016/j.prevetmed.2004.12.002 15748757

[B32] http://issuu.com/harvardpublichealth/docs/hph_winter2012 Singer T: **The debate heats up: raw milk.** *Harvard Public Health Rev* 2012, 22-23,29. .

[B33] Langer AJ Ayers T Grass J Lynch M Angulo FJ Mahon BE Nonpasteurized dairy products, disease outbreaks, and state laws-united states, 1993–2006 Emerg Infect Dis 2012 18 3 385 391 http://wwwnc.cdc.gov/eid/article/18/3/pdfs/11-1370.pdf http://wwwnc.cdc.gov/eid/article/18/3/pdfs/11-1370.pdf 10.3201/eid1803.111370 22377202 PMC3309640

[B34] http://www.aphis.usda.gov/traceability/downloads/report_implementation_plan.pdf United States Department of Agriculture/Animal and Plant Health Inspection Service: **Animal disease traceability framework, comprehensive report and implementation plan.** 2011, .

[B35] Traceability for livestock moving interstate Fed Regist 2013 78 6 2040 2075 http://www.regulations.gov/#!documentDetail;D=APHIS-2009-0091-1627 http://www.regulations.gov/#!documentDetail;D=APHIS-2009-0091-1627

